# Language Development in Early Childhood: Quality of Teacher-Child Interaction and Children’s Receptive Vocabulary Competency

**DOI:** 10.3389/fpsyg.2021.649680

**Published:** 2021-07-15

**Authors:** Ning Yang, Jiuqian Shi, Jinjin Lu, Yi Huang

**Affiliations:** ^1^School of Education, South China Normal University, Guangzhou, China; ^2^Kindergarten Affiliated to Jinan University, Guangzhou, China; ^3^School of Foreign Languages, China University of Geosciences, Wuhan, China; ^4^Department of Psychology, Institute for Research of Children, Youth and Family, Faculty of Social Sciences, Masaryk University, Brno, Czechia

**Keywords:** teacher-child quality interaction, Chinese children, receptive vocabulary development, early years, modeling

## Abstract

High-quality teacher-child interactions in early learning environments have been regarded as a key contributor to children’s early language and cognitive development in international scholarships. Little is known, however, about the longitudinal effects of children’s receptive vocabularies in the Chinese context. In this study, we addressed the question of such longitudinal effects by examining the predictive effect of preschool teacher-child interaction quality on children’s subsequent receptive vocabulary development in 42 kindergartens in Guangdong Province China. The results in a nested design showed that except for the factor of Emotional Support, the other two factors (Classroom Management and Instructional Support) were positive predictors to children’s vocabulary competency from K2 (T1) to K3 (T2) at preschools. Findings contribute to the growing international literature on the critical role teacher-child interaction quality plays in children’s language and literacy learning and development. Implications for enhancing communication channels between early childhood (EC) educators and decision-makers, and the strategies of the improvement of language and literacy teachers’ professional development are also discussed.

## Introduction

Vocabulary is critical for children to develop language, literacy, and communication in their early years. Children with poor language skills, particularly in terms of poor receptive language, are likely to have low school readiness and are at risk for subsequent academic problems (NICHD Early Child Care Research Network, 2005). Previous studies have shown that if children have high-quality preschool experiences, their learning and cognitive development will be promoted concurrently and longitudinally (Shonkoff and Phillips, 2000; Reynolds et al., 2011; Campbell et al., 2014; Yoshikawa et al., 2015). To date, these empirical studies have been dominantly undertaken in the United States and a number of developed countries in Europe (NICHD Early Child Care Research Network, 2005; Pianta et al., 2008; Curby et al., 2009; Sylva et al., 2011; Anders et al., 2012). In addition, scholars (Hoff, 2006; Pianta, 2006; Dickinson and Porche, 2011) believe that children spend the most time with teachers in their classrooms, so teachers’ language interaction and communications could become unique sources for language input in children’s daily lives.

However, in China, very few studies have focused on the impacts of teacher-child interaction quality on the development of children’s receptive vocabulary and communication longitudinally, particularly in the case of left-behind children. According to the China National Committee of Family Planning, there were approximately 9.02 million Chinese left-behind children in 2016 (Wang and Zhao, 2016). National Bureau of Statistics (2020) shows that migrant workers’ income could reach 50,000 RMB per person every year if they work in big cities. However, if migrant workers choose to work in less developed areas, they might only obtain one-third of the income of those who work in big cities. In this case, most migrant workers would work in developed coastal provinces, such as Guangdong and Zhejiang Provinces. When parents migrant to work in big cities (usually over 6 months), it is common that their children are left at home and taken care of by grandparents (Zhao et al., 2009).

Chinese left-behind children are more found in southern and central regions of China. Compared with children in Western countries, Chinese left-behind children are culturally unique due to the circumstances of social and economic development in mainland China. In contrast to western grandparents who take the responsibility of caring, Chinese grandparents’ rearing happened in a different context since economic reform launched in 1979 (Cox, 2008; Hayslip et al., 2017). Since the Chinese open-door policy, Chinese young parents have been moved from the less developed regions for seeking work opportunities in big cities, grandparents have to take care of children for saving money (Burnette et al., 2012). These Chinese grandparents lack formal adequate financial support and government or community support, which might lead to the lack of self-efficacy of raising children and well-being. In the home environment, grandparents could afford to provide necessities, but they cannot replace parents’ essential roles in children’s emotional and academic development (Burnette et al., 2012). They demonstrated less tolerance of children’s difficult behaviors and more authoritarian parenting than those in the United States (Wang et al., 2019).

The current study aimed to address this research gap by examining the impacts of teacher-child quality interactions on Chinese left-behind children’s receptive vocabulary development in K2 (4–5 years old) and K3 (5–6 years old) at preschools. Longitudinal examinations contribute to early childhood education (ECE) policy and provide decision-makers with evidence to enhance EC policy in China. Additionally, the empirical study provides first-hand data to EC educators to assist teachers in the development of their teaching style and service.

The teaching-through-interactions (TTI) method introduced by Pianta and Hamre (2009) provides a significant and practical framework to measure teacher-child and peer interactions in classrooms. The framework has been used widely in a global context (Hu et al., 2020; Suchodoletz et al., 2020). It is rooted in human development and ecological systems (Bronfenbrenner and Morris, 1998) and represents the dynamic and concurrent interactions that have short- and long-term positive effects on children’s academic and cognitive development. TTI not only suggests effective classroom practices (Hamre et al., 2013) but also recommends high-quality classroom interactions, which may provide evidence for the diagnosis of high-risk students and assist these students through early intervention practices (Hamre and Pianta, 2005). Moreover, the TTI framework helps teachers focus on specific content forms of support that aim at teaching particular skills and imparting knowledge to students in different subjects.

### Children’s Vocabulary Development in the Early Years

The preschool years have been regarded as the most important time for children to develop their vocabulary competency before joining formal learning at schools (Blocker, 2017; Grøver, 2017). Children’s early vocabulary development has a significant influence on their academic performance over the life span (Chall et al., 1990; Williford et al., 2013). For example, Christ and Wang (2010) pointed out a potential gap of 600 words among 3-year-old children with different family backgrounds and noted that the gap may enlarge when the children are enrolled in school. Similar research results were found by Dickinson and Porche (2011) and Vandell et al. (2016), who claimed that the achievement gap established at this age seems to persist into school and later life because of the rank-order stability in children’s development after they are 4 years old. Children’s vocabulary skills are often measured in terms of the comprehension of words (receptive vocabulary) and the production of words (expressive vocabulary) (Hoff, 2014). These two components are important to examine children’s speaking proficiency, including word knowledge, knowledge of word order, grammatical rules, and conceptual knowledge, which can predict their later reading ability (Storch and Whitehurst, 2002; Scarborough, 2009).

Children’s vocabulary development was examined in different social-cultural contexts (e.g., Taylor et al., 2013; Spilt et al., 2015; Hartman et al., 2017). Taylor et al. (2013) investigated a sample of 4,332 Australian children and found that socioeconomically disadvantaged regions, family background and children’s individual factors had significant influences on their receptive vocabulary comprehension in the growth model. A similar study was also undertaken in Australia by Spilt et al. (2015), who believed that close teacher-child relationships and frequent peer interactions were essential for children to develop language skills in their early years. In the United States, Hartman et al. (2017) showed that there was a positive correlation between mothers’ oral language ability and children’s receptive language outcomes. More recently, Hansen and Broekhuizen (2020) found that the early childhood learning environment (i.e., early childhood centers and preschools), quality of staff interactions and conversation support had a significant influence on young children’s language skills. According to a Chinese study (Hu et al., 2020), teachers’ emotional support has a close relationship with the vocabulary of children from 3 to 5 years of age.

In addition to the social context, parenting styles, quality of caregivers, parents’ education and family socioeconomic status were found to be associated with children’s language and cognitive development in the early years. These potential variables were considered conceptually important in prior studies of teacher–child interactions (i.e., Mashburn et al., 2008; Hamre et al., 2012; Cabell et al., 2013), Additionally, previous research suggests that family socioeconomic status incorporates family income and parental education in developing countries (Bollen et al., 2001). Moreover, the quality of the communication foundation that caregivers and children construct together includes the kinds of engaging and responsive interactions that contribute to language growth both in and out of school (Adamson et al., 2014; Hirsh-Pasek et al., 2015; Perry et al., 2018; Romeo et al., 2018). These family and personal attributes may be more important for left-behind children, as they have little support from parents (Guo et al., 2020).

### Teacher-Child Interactions and Children’s Vocabulary Development

Evidence from previous studies has primarily indicated that children’s vocabulary competency is closely related to teacher-child interactions in preschools (Weisleder and Fernald, 2013; Spilt et al., 2015; Sun and Verspoor, 2020; Sun et al., 2020). High quality of teacher-child interaction has been documented as a positive factor that impacts children’s receptive vocabulary acquisition (Dickinson and Porche, 2011; Gonzalez et al., 2014). In recent years, researchers have paid attention to the quality of teacher-child interactions in specific domains by using the Classroom Assessment Scoring System (CLASS) (Pianta et al., 2008) to measure pre-K classroom interaction quality. It is composed of ten subitems that evaluate teacher performance on a scale from 1 to 7 across three broad domains: emotional support, classroom organization, and instructional support.

These three factors were also found to have strong associations with children’s vocabulary development. For example, Curby et al. (2013) examined whether consistency in teachers’ emotional support (ES) is related to better receptive vocabulary competency among preschool children (averaged 4.6 years old). The results showed that children who obtained consistent emotional support at preschools had a higher level of language competency, social communication and academic outcomes than those who did not. Additionally, researchers (Curby et al., 2013) proposed that consistency needs to be considered when evaluating teachers’ emotionally supportive interactions. Supported results were found in some studies (Schmitt et al., 2012, 2014), which posited that the level of emotional support in preschool classrooms significantly predicted the language gain over the academic year. Schmitt et al. (2012) demonstrated that preschool children (4–6 years old) who were supported by teachers with high degrees of warmth and concern showed greater gains in language than those learning in an environment with low emotional support. A mixed result was found by Weiland et al. (2013), who showed very little correlation between the quality of teacher-child interactions and preschool children’s vocabulary and cognitive development. Also, supported studied were found by American (Perlman et al., 2016) and European researchers (Ulferts et al., 2019), who indicated that weak to null associations between the domains of teacher-child interactions and measures of children’s language and literacy skills.

The second dimension, classroom organization (CO), refers to “how teachers maximize learning efficiency and children’s engagement in classroom learning by proactively managing behavior and using rich and diverse learning modalities to motivate learning” (Hu et al., 2020, p. 3). How teachers organize the class and days at preschools contributes to the variety of young children’s language development (Dickinson et al., 2014), and the quality of teacher-child interaction skills predicts children’s class engagement, literacy development and academic achievement (Dobbs-Oates et al., 2011; Downer et al., 2012). More recently, Chinese researchers (Hu et al., 2020) found that teacher–children interaction quality, especially the in classroom organization domain, consistently predicted the development of preschool children’s early vocabulary skills. These studies showed that higher levels of preschool CO related positively to children’s emergent language development and academic achievement (Dobbs-Oates et al., 2011; Downer et al., 2012).

The last dimension of the CLASS, instructional support (IS), is believed to be the most important domain due to its close association with children’s receptive vocabulary development (Hu et al., 2017). Evidence has been found in different countries, such as Chile (Leyva et al., 2015), Germany (Suchodoletz et al., 2014), and China (Hu et al., 2017). Paulick (2019) showed that teachers who had higher scores on the dimension of “Instructional Support” of CLASS instrument in the study provided children with more opportunities to obtain more modeling of talk and more exposure to sophisticated vocabulary. Generally, teachers who have a higher score on IS like to ask open questions and provide immediate and constructive feedback to enhance children’s motivation, efforts, interests, creativity, and learning strategies, which would be beneficial factors for preschool children’s receptive vocabulary development (Pianta, 2006).

Moreover, recently, researchers have shifted their focus to the investigation of correlations between the quality of teacher-child interactions and the language development of children with English as their native language and children with non-English backgrounds (Gonzalez et al., 2014; Farkas, 2019; Langeloo et al., 2019). Gonzalez et al. (2014) found that children’s language and literacy ability were significantly influenced by the quality of teacher-child interactions in the preschool years. Similarly, Farkas (2019) examined the relationship between teachers’ emotional and cognitive competence and bilingual preschool children’s receptive language development in Chile. The results showed that teachers who have a higher emotional and cognitive competence represent an important predictor of children’s vocabulary development in the early years. It indicated that teachers’ affection and care are essential for preschool children who are not English native speakers. Langeloo et al. (2019) reviewed previous studies to demonstrate that the importance of high-quality teacher-child interaction in daily life could influence multilingual children in multicultural contexts. Drawing from the results of these empirical studies, we can conclude that the quality of teacher-child interaction is an important indicator of children’s vocabulary development in both English and non-English-speaking environments.

### ECEC in the Chinese Context

In China, ECEC is not a necessary component in the national 9-year compulsory education system, but it served more than 46 million children ages 3–6 in 2017, aiming to reach a target of 85 percent of Chinese children who could be enrolled in preschools in 2020 (Ministry of Education of the People’s Republic of China, 2014). Due to a lack of legislation in ECEC in China, both public kindergartens (monitored by government agencies) and private kindergartens (monitored by a local community or through the private sector as a for-profit program) exist. Generally, the two models follow the same preschool structures. That is, Chinese children go through 3 years of preschool education: K1 (3- to 4-year-olds), K2 (4- to 5-year-olds) and K3 (5- to 6-year-olds). Chinese kindergartens usually feature large class sizes and a high student-to-teacher ratio (Hu et al., 2016b). In such large classrooms, it might be very difficult for teachers to undertake a student-centered approach, and class management may not be as flexible as that in the Western context (Chan and Rao, 2010). In this case, principles and strict obedience requirements are the focus in daily teaching.

Chinese ECE teaching and learning have been heavily influenced by Chinese moral and cultural values (Hu et al., 2016a, b). Culturally, parents, preschool teachers and children are driven by a competitive and examination-oriented educational system to focus on academic learning outcomes at an early stage. In this case, children have very few opportunities to be supported in the development of their creativity, self-discovery, and problem-solving skills. Additionally, the unique Chinese cultural values, such as Confucianism, collectivism and Buddhism, lead teachers to instruct children to maintain harmony with others in a group and force them to adapt to the Chinese cultural value system (Tobin et al., 2009; Lu, 2019). In addition, large class sizes and the low qualification or experience of teachers can influence their interactions and relationships with young children. This situation might be more common in socioeconomically disadvantaged areas in China (United Nations Children’s Fund (UNICEF), 2019^1^; Hu et al., 2020). Given these unique sociocultural factors in the Chinese ECE system and considering the variation in teaching methods across the years of preschool education, the effect of teacher–child interactions on children’s language ability might vary across different preschool years. In this case, we aim to examine whether teacher-child interaction quality has a predictive role in children’s receptive vocabulary in two preschool years in the Chinese social context.

## Materials and Methods

### Participants

The present study is part of an ongoing national project that explores the correlation of teacher-child interaction quality with Chinese left-behind children’s early development. The project was undertaken in Guangdong Province, China. We selected the province for two main reasons: (1) The province holds the largest number of families with different socioeconomic statuses, and (2) it has broad urban-rural disparity due to the presence of many migrant workers and families. Considering these characteristics, participants were selected using a stratified, random sampling approach. First, based on the Guangdong government’s recent economic report, three economic levels (advanced, average, and below average) were identified. Then, 20 preschools were randomly selected at the three economic levels, respectively, from the list of preschools provided by each municipality’s local education committee. Within each preschool, we randomly selected one K2 classroom (the second year of preschool) to be included in the sample. Finally, ten children in each classroom at the chosen preschools were randomly selected to participate in the longitudinal study. However, seven preschools in the below-average economic municipality closed in the second year, and six preschools in the high-economic municipality and four in the average economic municipality dropped out due to other administrative commitments in the second year. Technical issues were found in one preschool in the average economic municipality during our visit in the second year. In the end, a total of 42 classrooms in 42 different preschools were included in this study, of which nine classrooms (21%) were part of public programs. The children in the schools that dropped out and had technical issues were excluded from the current study.

This longitudinal study was initiated in May 2015 when the children were in their second year of preschool (T1). Then, data were collected in May of 2016 (T2). Only teachers who participated in the national project (*N*_*female*_ = 354) were considered for inclusion in the present study. Of these teachers, we included only teachers who had finished novice^2^ teachers’ training and obtained their registration in the sampled preschools (*N*_*female*_ = 70). Of the 70 teachers (*M*_*female*_ age = 27.3) included in these analyses, 51 (72%) teachers had received only one form of professional development aimed at improving their interactions with children. Teachers had an average of 2.8 years of experience teaching preschool-age children and were homogenous in terms of educational backgrounds (BA degree = 66, diploma/certificate = 4). All preschool teachers had an education degree, certificate or diploma of early childhood education. All preschool teachers were of Han nationality according to their identification cards in China.

The children enrolled in these 42 participating classrooms had an average age of 4.29 years old (SD = 0.59). In the sampled preschools, the average class size was 10, and the teacher-children ratio was 1:6, on average (see Table 1). All children were of Han nationality. The final sample of left-behind children included 181 girls and 173 boys, whose parents were not with them for at least 6 months during the period of the experiment. In daily life, all of the children were cared for either by their grandparents or guardians, and they were healthy during the study.

**TABLE 1:** Descriptive statistics of the variables.

Variables		*N*	Min	Max	Mean	SD	Chi-square (wave 1)	Chi-square (wave 2)
**Verbal ability**								
	• PPVT T1	354	3	106	40.4	20.3		
	• PPVT T2	354	4	105	51.0	22.2		
**CLASS**								
	• Positive climate T1	354	3.25	6.05	5.46	0.77		
	• Negative climate T1	354	1.00	3.00	1.31	0.45		
	• Teacher sensitivity T1	354	2.75	6.00	4.32	0.83		
	• Regard for student perspective T1	354	2.75	5.75	4.25	0.78		
	• Behavior management T1	354	3.00	6.75	5.44	0.93		
	• Productivity T1	354	3.25	6.25	4.90	0.82		
	• Instructional learning formats T1	354	2.50	5.25	4.11	0.78		
	• Concept development T1	354	1.25	4.00	2.75	0.57		
	• Quality of feedback T1	354	1.50	4.00	2.67	0.64		
	• Language modeling T1	354	1.75	4.00	2.75	0.57		
	• Positive climate T2	354	3.75	7.00	5.41	0.83		
	• Negative climate T2	354	5.00	7.00	6.80	0.44		
	• Teacher sensitivity T2	354	2.75	6.25	4.42	0.94		
	• Regard for student perspective T2	354	2.00	6.25	4.31	0.99		
	• Behavior management T2	354	3.75	7.00	5.47	0.84		
	• Productivity T2	354	3.25	6.50	5.29	0.73		
	• Instructional learning formats T2	354	3.00	5.75	4.51	0.87		
	• Concept development T2	354	1.00	4.50	2.40	0.75		
	• Quality of feedback T2	354	1.25	5.00	2.64	0.79		
	• Language modeling T2	354	1.50	4.75	2.80	0.94		
Additional covariates		N	Frequency%					
**Location**							302.02**	283.84**
	• Developed big cities	131	37					
	• Less developed towns	115	32.5					
	• Rural/regional areas	108	30.5					
**Gender**							82.14	94.23
	• Male	173	48.9					
	• Female	181	51.1					
**Family annual income (RMB)**							221.70**	226.78*
	• Less than 50,000	112	31.6					
	• 50,000–100,000	67	18.9					
	• Over 100,000	175	49.4					
**Mothers’ education**							309.76**	336.48**
	• Completion of compulsory education	97	27.4					
	• High school graduates	72	20.3					
	• Diploma	88	24.9					
	• Bachelor Degree	97	27.4					
**Fathers’ education**							318.79**	349.64**
	• Completion of compulsory education	84	23.7					
	• High school graduates	88	23.7					
	• Diploma	58	16.4					
	• Bachelor Degree	124	35					
**Teaching experience**							72.93	85.90
	• Less than 5 years	66	94.2					
	• Over 20 years	4	5					

### Procedure

#### Recruitment Process

The ethical application was approved by the first author’s working institution in 2014. Before data collection, we approached the heads of the selected preschools to seek their for participation in the research project. With the assistance of the preschool teachers, every parent in the preschool was sent the research packet, which included research information, a flyer and consent forms. If the parents agreed to participate in the project, they completed forms covering basic family demographic information and consent forms. For convenience, they were allowed to return the packets either directly to the principal researcher via prepaid confidential envelopes or to the mailbox of the head of the preschool. The parents’ response rate was high at 98%.

#### Training and Coding for CLASS

Before conducting the classroom observation, eight research assistants (RAs) in psychology and education participated in a 4-day training on the CLASS. The training was organized by *TeachStone*^3^, which introduced the theoretical framework of the CLASS and provided detailed examples of coding procedures. After the workshop, all the RAs passed the online test to become qualified as certified raters using the CLASS.

#### Observation and Coding

Teacher–child interaction quality was assessed in May 2015 and May 2016. In May 2015 (T1), after the parents returned the survey, the eight research assistants videotaped teacher-child interactions in each of the 42 participating classrooms during the first 3 h of a typical day. After obtaining a 3-h video in each preschool classroom, the researchers extracted five 20-min video clips (i.e., observation cycles) for each classroom. To show a typical day in Chinese preschools, we collected the five typical routines and activities in cycles, such as group teaching, free play, outdoor activities, and lunch and tea breaks. Every coder made a final rating for 10 dimensions based on the scoring criteria in the CLASS manual (Pianta et al., 2008). We adopted the double-coding method to rate the quality of teacher–child interactions by dividing the research assistants, certified CLASS raters, into four pairs (Hu et al., 2020). According to Fan and Sun (2014), average interrater reliability estimates and an intraclass correlation coefficient (*ICC*) were calculated. The interrater consistency *ICC*s of the CLASS dimensions ranged from 0.82 to 0.94.

In 2016, the RAs undertook classroom observations during the spring of the preschool year that began in February. Four observation cycles per classroom were distributed throughout the morning, including the same activities videotaped in 2015. The observation cycles were 20 min long, on average. This added to a total of 80–85 min of observations per teacher and classroom. Classrooms were observed directly by the RA-trained CLASS observers in 2015, each of whom passed a reliability test prior to data collection and participated in regular training sessions (eight sessions with an average reliability of 95%).

### Instruments

#### Teacher–Child Interaction

The Classroom Assessment Scoring System (CLASS pre-K) was used to measure the teacher-child interaction quality (Pianta et al., 2008). This tool has demonstrated good reliability and validity in various countries (e.g., Cadima et al., 2010; Suchodoletz et al., 2014; Leyva et al., 2015). In China, Hu et al. (2016a) evidenced the applicability of the three domains in the CLASS to assess teacher-child interaction quality: emotional support (ES), classroom organization (CO), and instructional support (IS). There were 10 dimensions within the three domains. The dimensions of the CLASS were assessed on a Likert scale ranging from 1 to 7, with 1 or 2 indicating low levels of quality, 3, 4, or 5 indicating medium levels, and 6 or 7 indicating high levels.

#### Receptive Vocabulary

The Chinese version of the Peabody Picture Vocabulary Test–Revised (Lu and Liu, 2005) was adopted to measure children’s receptive vocabulary ability. The Chinese version is an adaptation of the Peabody Picture Vocabulary Test–Revised (Dunn and Dunn, 1981) and is used for 3- to 12-year-old children. The Chinese version has a high degree of reliability and validity and has been used in many studies (Cheng et al., 2009). The children were shown a card with four pictures, and the RAs read a word and asked the individual child to point to the picture that matched the word. Cronbach’s alpha for the scale was 0.93 in the current study.

Few missing data existed in this study (less than 0.4%). Little (1988) MCAR test indicated that these missing data were completely random (*X*^2^ = 117.03, *df* = 94, *p* > 0.05). As the number of missing data points was so small, we did not include them in the two waves (T1 and T2).

### Data Analysis

#### Descriptive Statistics

Descriptive statistics were run in the first step to summarize the descriptive results of each measurement. Additionally, the chi-square test was undertaken to investigate the differences in PPTV in the two waves among children with diverse demographic characteristics. In the further regression model, we only included factors that were significant in the chi-square test. All statistical procedures were conducted by SPSS 25.0 (see Table 1).

#### Psychometric Analysis

As CLASS was used to measure teacher-child interaction, it was necessary to first confirm its structure. First, we conducted confirmatory factor analysis (CFA) to reach the best conceptual construct of CLASS by using the “lavaan” package in R. The three frameworks were one-factor, two-factor and three-factor structures. We only adopted CLASS data from the first wave (T1) because the aim of the current study was to examine the prediction of CLASS regarding children’s language outcomes in the future. After selecting the best framework by comparing the above three CFA models, we continued to delete certain items according to the value of the factor loading and modification incidence.

#### Multilevel Modeling

We adopted multilevel analysis because of the hierarchical characteristics of the data, where teacher-child interactions were observed within preschools in different locations, which means that the variables of teacher-child interaction and children’s PPTV were nested in the location variable. The multilevel strategy based on the nested design has been adopted in many previous studies (e.g., Fukkink et al., 2017; Bachman et al., 2018; Hu et al., 2020). These studies enhanced our understanding of how children’s engagement and teachers’ interactions combine to predict school readiness and students’ academic outcomes. Thus, teacher-child interactions were seen as predictors of children’s receptive vocabulary in level 1, and the locations were entered into level 2. The preschool locations were grouped into three categories: (1) large cities; (2) medium-sized towns; and (3) regional areas. In addition, the planned control variables in level 1 were children’s gender, families’ socioeconomic status, parental education level (mother), and teachers’ teaching experience. However, if a planned control variable was not significant in the initial chi-square test, it was not entered into the model. The analysis was performed using the “lme4” package based on the maximum likelihood method.

## Results

Table 1 shows the descriptive statistics for all variables. In general, on the 7-point CLASS scale, teacher–child interaction quality ratings were approximately in the medium to low range of quality. At T1, the mean emotional support score of 3.84 and classroom organization score of 4.82 were well within the range for medium quality, which suggests that children experienced moderate levels of emotional support and classroom organization. However, the mean instructional support rating of 2.72 indicated that children experienced low levels of instructional support. At T2, although the mean scores of emotional support (mean = 5.24) and classroom organization (mean = 5.09) increased, the mean IS (mean = 2.61) rating was lower than that at T1. The chi-square tests indicated that there were no gender differences in PPTV in the two waves (T1 and T2), and likewise, PPTV in the two waves did not vary across different levels of teaching experience. Thus, those two variables were not included for later analysis.

According to the study undertaken by Hu et al. (2016b), there are three possible structures of CLASS: (1) all items belong to one factor; (2) only the factors of social support and instructional support are included; or (3) a three-factor structure including emotional support, classroom organization, and instructional support is possible. The information of model fit is shown in Table 2. The results suggest that the three-factor model is most suitable for this study.

**TABLE 2:** Indexes of model fit for three models.

	AIC	BIC	χ(df)	CFI	TLI	SRMR
One-factor	7937.02	8014.40	923.68 (35)	0.71	0.62	0.10
Two-factor	7729.50	7810.75	714.17 (34)	0.78	0.70	0.10
Three-factor	7661.39	7750.38	642.05 (32)	0.80	0.72	0.09

Although CFA suggested the three-factor model, all the items in CLASS contributed adequately to the matched factor except the item of negative climate, with a factor loading below 0.4 (0.37). After the deletion of the negative climate item, the model fit improved but was not yet ideal (CFI = 0.84, TLI = 0.96, SRMR = 0.09). The modification incidence suggested that there should be some connections of errors between observed variables and latent variables to reach a good model fit (CFI = 0.97, TLI = 0.92, SRMR = 0.06). In detail, the error of teacher sensitivity correlated with the errors of latent classroom organization, latent instructional support, and observed productivity. The error of the item observed regard for student perspective was correlated with the errors of latent classroom organization, latent instructional support and observed language modeling. The error of language was correlated with the observed variables of productivity and positive climate. In addition, the errors of positive climate and productivity were correlated. Next, we conducted Pearson correlation analysis based on the extracted latent variables of three factors in CLASS. The results are shown in Table 3.

**TABLE 3:** The results of correlations of teacher-child-interaction level variables and latent factors in CLASS.

	SES	edu_mother	edu_father	ES	CO	IS	PPVT_1	PPVT_2
SES	–							
edu_mother	0.61**	–						
edu_father	0.59**	0.82**	–					
ES	0.43**	0.48**	0.49**	–				
CO	0.39**	0.41**	0.42**	0.95**	–			
IS	0.45**	0.56**	0.58**	0.81**	0.68**	–		
PPVT_1	0.49**	0.54**	0.53**	0.48**	0.43**	0.52**	–	
PPVT_2	0.45**	0.50**	0.51**	0.43**	0.38**	0.52**	0.75**	–

SES, socioeconomic status of children’s families; Edu-mother, mother’s education level; edu-father, father’s; Exp, teacher’s experience in teaching. ES, CO, and IS were “emotional support,” “classroom organization,” and “instructional support” in CLASS, respectively. PPVT_2 referred to children’s scores of the second time on the Peabody Picture Vocabulary Test. *p < 0.05, **p < 0.01.

Finally, using the estimated latent values of three factors of CLASS, we conducted a multilevel regression model to investigate the impact of CLASS in wave 1 on the PPTV in wave 2. At the level centered on teacher-child interaction, the covariance variables were family socioeconomic status, mother’s education level, father’s education level, and the scores on the first PPVT test. The cluster was the location, which indicated regional economic development. The adoption of a random intercept model with a fixed slope showed that after the elimination of the effect of location, the factors of class organization and instructional support positively predicted children’s receptive vocabulary. The factor of emotional support was a negative predictor (see Table 4).

**TABLE 4:** A multilevel regression model.

	Estimate	Std. error	*t-*value
**(Intercept)**	22.12728	3.77946	5.855
**ES_1_sd**	−3.90756	5.00502	−0.781
**CO_1_sd**	1.22419	4.24528	0.288
**IS_1_sd**	5.13399	1.92647	2.665
**SES**	0.55682	1.13530	0.490
**edu_mother**	0.68726	0.93049	0.739
**PPVT_1**	0.63623	0.04954	12.843

## Discussion

Based on human development and ecological systems (Bronfenbrenner and Morris, 1998), teacher-child relationships play a pivotal role in children’s language and literacy development. This study is one of the few to examine correlations between the quality of teacher-child interactions and the receptive vocabulary development of children between 3 and 6 years of age in the Chinese sociocultural context in a dynamic way. This study indicates that children’s receptive vocabulary development in the early years is closely related to the quality of teacher-child interactions. The findings support and extend those of previous studies that have found a high quality of teacher-child interaction to be a predictor of developmental changes in receptive vocabulary development in early childhood (Reynolds et al., 2011; Spilt et al., 2015; Yoshikawa et al., 2015; Hu et al., 2016b; Farkas, 2019) and confirm processes observed in previous studies demonstrating that teachers develop closer relationships with children with higher language abilities (Justice et al., 2008). The findings show that after considering sociodemographic factors, such as gender, family SES, and parents’ education levels, the quality of teacher–child interactions Chinese children experience within a preschool has important and lasting effects. The details of the findings are discussed in the following parts.

### Classroom Organization and Instructional Support: Key Roles in Children’s Receptive Vocabulary Development

When we examined how teacher–child interaction quality in Chinese preschool classrooms predicted children’s later receptive vocabulary development, both classroom organization and instructional support emerged as important contributors. Classroom organization and instructional support quality consistently predicted children’s receptive vocabulary development in the K2 and K3 stages. Their positive correlations with children’s vocabulary development in the early years provided supportive evidence of previous study findings that children who experience high-quality classroom organization tend to have better language, literacy and academic performance (Dobbs-Oates et al., 2011; Downer et al., 2012; Dickinson et al., 2014). Classroom organization recognizes the importance of the management and teaching strategies used in the classroom. This study demonstrates that in highly organized classrooms, teachers adopt appropriate teaching strategies and create meaningful plays that can increase children’s opportunities to engage in social communication and contribute to their language and academic development.

Similarly, this study found that instructional support quality predicted children’s receptive vocabulary scores in the following year. In other words, in classrooms with higher-quality instructional support, teachers use more constructive feedback and support to promote children’s higher-order thinking and cognitive language skills, and this is highly related to the development of the children’s language and communication skills in the following year. This result does not support Weiland et al. (2013) finding that classroom quality had a small to null correlation with children’s cognitive development. The inconsistent results might be due to different ethnics, and data collection intervals in two studies. In Weiland’ study (2013), young children were from nine ethnics and their receptive vocabulary competency was scored in spring and fall semesters in the same year rather than in two consecutive years. However, the study results corroborate many previous studies showing that teachers’ frequent talk, instructional support in reading and time spent after reading were closely related to children’s receptive vocabulary development (e.g., Weisleder and Fernald, 2013; Gonzalez et al., 2014; Spilt et al., 2015).

Chinese children usually enter a formal preschool setting at the age of 3 (Ministry of Education of the People’s Republic of China, 2020). Compared with children with parents, Chinese left-behind children feel more vulnerable, so teachers need to consider providing them with close attention and gentle care in preschools (Chang et al., 2019). In the absence of an effective and supportive home learning environment, well-organized classroom activities and effective support become more important attributes to increase the interaction opportunities between teachers and peers and, as a consequence, to improve children’s vocabulary proficiency (Hansen and Broekhuizen, 2020).

### Emotional Support: A Possible Different Role

In this study, we detected a negative correlation between emotional support quality and children’s receptive vocabulary development over a 2-year period. Our finding differs from that of multiple studies in which researchers found emotional support to be correlated with higher cognitive and language skills, including vocabulary, reading, and cognitive function (Hamre and Pianta, 2005; Curby et al., 2013; Hamre et al., 2013; Schmitt et al., 2014). Several other studies, however, also reported a lack of correlation between ES and academic (inducing vocabulary, reading, literacy, and numeracy) skills (Burchinal et al., 2008; Howes et al., 2008; Cadima et al., 2010). It is worthwhile to note that teachers showed a higher level of quality for ES in T1 and T2, with an average 4.7. This could be explained by two possible reasons. First, when Chinese young children met their basic needs for ES in K2, the contributions to children’s vocabulary development skills are limited in K3 (Hu et al., 2019). Second, although Chinese teachers’ mean scores are high in ES in terms of the warmth and safety they provide to children, they tend to score low on language modeling, providing effective feedback and promoting children’s interest in sharing their ideas. Without cultivating children’s interest, Chinese teachers usually assigned books and reading materials that they believed beneficial for children’s vocabulary development. Also, teachers usually are the central in the group activities rather than children. All these restricted activities might not play a beneficial role in the preschool children’s vocabulary development. This has been evidenced by Vitiello et al. (2012), who showed improvement over the school year in measures of students’ language and literacy when they were in classrooms with lower (vs. higher) instructional and emotional support. Rudasill et al. (2016) found similar results in preschools. Moreover, a large number of left-behind children are sent to preschools in medium- and less-developed areas rather than to preschools in developed urban areas. Previous studies found that young children with poor social-emotional development tend to have poor language skills (Stewart et al., 2005), and these challenges increase for young children who are vulnerable, as they tend to have more language and literacy difficulties than their peers (Phillips et al., 2008). Left-behind children from low-income families might be more risky of having less language compared to their higher income peers (Song et al., 2014). A lack of quality of caregiver-child conversations might be influential for children’s language development in a long term (Hirsh-Pasek et al., 2015). In this regard, teachers might consider to strengthen their efforts to provide meaningful emotional support for left behind children.

Another reason for the low level of association between emotional support quality and children’s receptive language development might be that preschool teachers tend to score low on promoting children’s interests and independence in the development of their communication skills (Hu et al., 2017). Influenced by Confucianism, left-behind children need to learn how to live and learn in a harmonious environment, which is an important task for teachers in their routine. In this case, children are not provided with many opportunities to make choices for themselves, and consequently, some children who are shy and introverted might not be brave enough to talk, read and write because they are afraid of being recognized as “naughty students” (Lu, 2019). Additionally, teaching with a whole-group approach is likely to decrease children’s interest in sharing ideas, talking with teachers and peers and showing curiosity in reading and writing. As a result, the lack of linguistic understanding and quality interaction plays a negative role in left-behind children’s receptive vocabulary development.

## Conclusion, Limitations, and Implications

This preliminary study has explored the correlations of teacher-child interaction quality and children’s receptive vocabulary development in a group of Chinese left-behind children and yielded meaningful results. First, we found that the three-factor structure of the CLASS was suitable for this Chinese cohort. Second, high-quality classroom organization and instructional support could positively predict left-behind children’s receptive language development in the following year. However, the quality of emotional support was negatively predictive of left-behind children’s receptive language development.

This study has several limitations. First, we conducted the psychometric analysis for CLASS based on a limited sample. The sample size could be enlarged to be more representative of the Chinese context. In the design, we did not include the school ID nested in different locations because of the tight research timeline and limited budget, which prevented the use of three-level data analytic strategies. The use of such strategies could yield more meaningful and interesting results. Second, the preschool teachers who participated in the study were almost all female novice teachers with less than 5 years of teaching experience in the sampled preschools. This might be a reason for the low mean ES scores in the study. Although female teachers are dominant in Chinese preschools, researchers could invite more male preschool teachers and educators to join in research projects. The gender differences between the two groups of teachers might be another interesting aspect to consider in the quality of teacher-child interaction. Third, we only assessed children’s receptive vocabulary development twice, from the second year to the third year of preschool, because of the budget limitations. In future research, more longitudinal studies may cover children’s language development trajectory from the first year to the last year of preschool in a growth model. Fourth, this study was undertaken in a southern province with a large number of migrant workers. The preschool teachers and children in this province may behave differently than those in other Chinese provinces. Fifth, we only examine Han nationality left-behind children’s receptive vocabulary. Future researchers could include more language domains and more background of children’s language and literacy environment. Last, due to the limited research budget, we did not have access to Children’s home language and literacy environment (e.g., language exposure at home, and reading frequency per week), which could influence children’s early receptive vocabulary learning (Sun, 2019).

Nevertheless, this study offers important implications for Chinese left-behind children and preschool teachers. Preschool teachers need to consider promoting left-behind children’s social development skills with an enhanced understanding of the children’s mental and psychological aspects. For example, teachers need to make an effort to provide more social interaction opportunities for left-behind children to communicate with others. Language stimulation and frequent peer interactions could help left-behind children develop their oral language skills and mental health (Spilt et al., 2015). In addition, more professional development programs need to be provided to young preschool teachers to further reinforce recent new initiatives on promoting child-centered teaching and learning. Most importantly, one-on-one feedback should be encouraged to help teachers use more child-centered practices in the Chinese context.

## Data Availability Statement

The raw data supporting the conclusions of this article will be made available by the authors, without undue reservation.

## Ethics Statement

The studies involving human participants were reviewed and approved by the China South Normal University. The patients/participants provided their written informed consent to participate in this study.

## Author Contributions

NY made substantial contributions to the data analysis and interpretation, conception and design of the article, drafting the article, and revising it critically. JS made substantial contributions to the data analysis and interpretation, and drafting and revising the article. YH collected the data and contributed to the data analysis. JL made substantial contributions to the conception and design of the study, and critically revised and approved the final version to be published. All authors certify that they have participated sufficiently in the work.

## Conflict of Interest

The authors declare that the research was conducted in the absence of any commercial or financial relationships that could be construed as a potential conflict of interest.

## Footnotes

1 According to the United Nations Children’s Fund (UNICEF), 2019 Annual Report, there are approximately 69 million children left behind due to migration, which is equivalent to thirty percent of the children in rural area. The number of left behind children is unevenly distributed across age groups, regions, and gender. The majority of the left-behind children population is located in south and central regions of China. In China, left-behind children are educationally and mentally disadvantaged due to lack of parents’ love and care. After migration, the family annual income would be depended on parents’ jobs and locations.

2 In the article, novice teachers have less than 5 years of teaching experience, and experienced teachers have at least 20 years of EC experience in China (Curry et al., 2016).

3 www.teachstone.org
